# Sodium intake according to NOVA food classification in Brazil: trends from 2002 to 2018

**DOI:** 10.1590/0102-311XEN073823

**Published:** 2024-02-26

**Authors:** Eduardo Augusto Fernandes Nilson, Giovanna Calixto Andrade, Rafael Moreira Claro, Maria Laura da Costa Louzada, Renata Bertazzi Levy

**Affiliations:** 1 Programa de Alimentação, Nutrição e Cultura, Fundação Oswaldo Cruz, Brasília, Brasil.; 2 Núcleo de Pesquisas Epidemiológicas em Nutrição e Saúde, Universidade de São Paulo, São Paulo, Brasil.; 3 Escola de Enfermagem, Universidade Federal de Minas Gerais, Belo Horizonte, Brasil.; 4 Faculdade de Saúde Pública, Universidade de São Paulo, São Paulo, Brasil.; 5 Faculdade de Medicina, Universidade de São Paulo, São Paulo, Brasil.

**Keywords:** Sodium, Food Habits, Nutrition Policy, Nutrition Surveys, Sódio, Hábitos Alimentares, Política Nutricional, Inquéritos Nutricionais, Sodio, Hábitos Alimentarios, Política Nutricional, Encuestas Nutricionales

## Abstract

Excessive sodium intake is a major global public health issue and the identification of dietary sources and temporal trends in its consumption are a key to effective sodium reduction policies. This study aims to update estimates of sodium intake and its dietary sources in Brazil according to the NOVA food classification system. Records of 7-day food purchases of households from the *Brazilian Household Budgets Survey* of 2002-2003, 2008-2009, and 2017-2018 were converted into nutrients using food composition tables and the mean availability was estimated per 2,000kcal/day. Mean daily sodium available for consumption in Brazilian households has increased from 3.9 to 4.7g per 2,000kcal, from 2002-2003 to 2017-2018, over twice the recommended levels of sodium intake. From 2002-2003 to 2017-2018, the processed culinary ingredients, including table salt, represented the largest dietary source of sodium, although their participation in dietary sodium was reduced by 17% (66.6% to 55%), while the percentage of dietary sodium from processed foods increased by 20.3% and from ultra-processed foods increased by 47.6% (11.3% to 13.6% and 17% to 25.1%, respectively). In conclusion, the total household sodium availability remains high and has increased over time in Brazil, yet the participation of different dietary sources of sodium have gradually changed.

## Resumo

A ingestão excessiva de sódio é um dos principais problemas de saúde pública em todo o mundo e a identificação de fontes alimentares e tendências temporais no seu consumo são fundamentais para a elaboração de políticas eficazes de redução de sódio. Este estudo tem como objetivo atualizar as estimativas de ingestão de sódio e suas fontes alimentares no Brasil de acordo com o sistema de classificação NOVA. Os registros de compras de alimentos no período de 7 dias de famílias das *Pesquisas de Orçamentos Familiares* de 2002-2003, 2008-2009 e 2017-2018 foram convertidos em nutrientes utilizando tabelas de composição de alimentos. A disponibilidade média foi estimada em 2.000kcal/dia. A média diária de sódio disponível para consumo nos domicílios brasileiros aumentou de 3,9 para 4,7g por 2.000kcal, de 2002-2003 a 2017-2018, mais do que o dobro dos níveis recomendados de ingestão desse nutriente. De 2002-2003 a 2017-2018, os ingredientes culinários processados, incluindo o sal de cozinha, representaram a maior fonte de sódio, embora a sua participação no sódio dietético tenha sido reduzida em 17% (de 66,6% para 55%), enquanto a porcentagem de sódio dietético dos alimentos processados aumentou 20,3% e dos alimentos ultraprocessados aumentou 47,6% (11,3% para 13,6% e 17% para 25,1%, respectivamente). Concluindo, a disponibilidade total de sódio nos domicílios permanece alta e tem aumentado ao longo do tempo no Brasil, mas a participação de diferentes fontes dietéticas de sódio mudou gradualmente.

## Resumen

La ingesta excesiva de sodio es uno de los principales problemas de salud pública en todo el mundo, y la identificación de las fuentes alimentarias y tendencias temporales en su consumo son esenciales para desarrollar políticas efectivas de reducción de sodio. Este estudio tiene como objetivo actualizar las estimaciones de la ingesta de sodio y sus fuentes alimentarias en Brasil según el sistema de clasificación NOVA. Los registros de compras de alimentos en el período de 7 días de familias de las *Encuestas de Presupuestos Familiares* de Brasil de 2002-2003, 2008-2009 y 2017-2018 se convirtieron en nutrientes utilizando tablas de composición de alimentos. La disponibilidad media se estimó en 2.000kcal/día. El promedio diario de sodio disponible para el consumo en los hogares brasileños aumentó de 3,9 a 4,7g por 2.000kcal, entre 2002-2003 y 2017-2018, más del doble de los niveles de ingesta recomendados de este nutriente. Entre 2002-2003 y 2017-2018, los ingredientes culinarios procesados, incluida la sal de mesa, representaron la mayor fuente de sodio, aunque su participación en el sodio dietético se redujo en un 17% (del 66,6% al 55%), mientras que el porcentaje de sodio dietético de los alimentos procesados aumentó un 20,3% y de los alimentos ultraprocesados aumentó un 47,6% (11,3% a 13,6% y 17% a 25,1%, respectivamente). En conclusión, la disponibilidad total de sodio en los hogares sigue siendo alta y ha aumentado a lo largo del tiempo en Brasil, pero la proporción de diferentes fuentes dietéticas de sodio ha cambiado gradualmente.

## Introduction

Inadequate diets are major modifiable risk factors for noncommunicable diseases (NCDs) and globally are responsible for 11 million deaths and 255 million disability-adjusted life years (DALYs). Among the dietary risk factors, high sodium intake contributes to 3 million deaths and 70 million DALYs ^1^.

Most populations consume excessive sodium (above 2g per day, which is the maximum daily intake recommended by the World Health Organization - WHO) ^2^ ^,^ ^3^. Additionally, in Brazil, excessive sodium intake was associated with 47,000 deaths and BRL 622 million in costs to the Brazilian Unified National Health System (SUS, acronym in Portuguese), considering hospitalizations, outpatient care, and drugs for hypertension control ^4^.

Diets with high sodium intake are associated with increased blood pressure and risk of cardiovascular diseases and other outcomes, such as gastric cancer, chronic kidney disease, kidney stones, and osteoporosis ^5^. The reduction of sodium intake is associated with reducing both systolic and diastolic blood pressure in hypertensive and normotensive individuals ^6^. Based on the current evidence and acknowledging the excessive sodium intake in most countries worldwide, the WHO recommends that sodium intake by adults should not exceed 2,000mg/day ^7^.

In 2013, using urinary sodium excretion, as part of the *Brazilian National Health Survey* (PNS, acronym in Portuguese), average sodium intake in Brazilian adults was estimated at 3,740mg/day, which is almost twice the WHO recommendation ^8^. Despite the excessive sodium intake by most of the population, only a small percentage of adults perceived their consumption as high and most people believed to consume adequate or even low amounts of salt ^9^.

Previous indirect estimates in Brazil, based on household food acquisition data from the *Brazilian Household Budgets Survey* (POF, acronym in Portuguese) in both 2002-2003 and 2008-2009, estimated an average sodium intake of 4.7g/day ^10^ ^,^ ^11^. From 2002-2003 to 2008-2009, the major dietary sources of sodium for the Brazilian population were still table salt added to foods and salt-based condiments (reduced from 76.2% to 74.4%), followed by industrialized foods and ready meals (increased from 17.2% to 20.5%) ^11^.

Other indirect estimates comparing food records and recall data from the POFs, found that over half of the sodium in the diet of Brazilians originate from breads, rice, beans, legumes, and meats. They also found that daily sodium intake was slightly reduced from 2,529mg/day in 2008-2009 to 2,489mg/day in 2017-2018 ^12^.

Nevertheless, none of these studies considered the NOVA system to classify the dietary sources of sodium. The NOVA system classifies foods according to the level and purpose of food processing and considers four food groups: unprocessed (or natural) and minimally processed; culinary ingredients; processed foods; and ultra-processed foods ^13^.

This analysis is particularly important since the diet of Brazilians is still mostly based on unprocessed and minimally processed foods, although it has significantly changed with a remarkable increase in the consumption of ultra-processed foods over the last decades. Notably, ultra-processed foods have a poor nutrient profile, including high sodium, fat, and sugar contents, and have been associated with many noncommunicable diseases ^14^.

This study aims to update the estimates for sodium intake and its dietary sources in Brazil, analyzing time trends in their participation according to the NOVA food classification.

## Methods

This study used data from the POF, conducted by the Brazilian Institute of Geography and Statistics (IBGE) from June 2002 to July 2003, May 2008 to 2009, and July 2017 to July 2018 ^15^ ^,^ ^16^ ^,^ ^17^.

The POF editions of 2002/2003, 2008/2009, and 2017/2018 employed a complex clustered sampling procedure, selecting census tracts during the first stage and households within those tracts during the second. Selection of census tracts was preceded by an examination of all tracts from the most recent *Demographic Census* to obtain strata of households with high geographic and socioeconomic homogeneity. For this classification were considered: the geographic location of tracts (region, state, capital city or other, urban, or rural) and, within each geographic locus, the variation, in years, of schooling of the head of the household in the sector (obtained during the *Demographic Census* or in the population count between censuses). Finally, about 500 strata of households that were geographically and socioeconomically homogeneous were constituted for each POF. The number of tracts selected from each stratum was proportional to the total number of permanent private households in that stratum. Then, households were selected within each tract by random sampling without reposition. Interviews within each selected stratum were distributed uniformly across the four trimesters of the study to reproduce, within each stratum, the seasonal variation in income and purchases.

For the nationally representative analysis: in 2002-2003, the total 48,747 households of the sample produced 443 strata with an average of 109.4 households per strata (ranging 9-801 households); in 2008-2009, the 55,970 households of the sample produced 550 strata with an average of 101.7 households per strata (ranging 8-796 households); and, in 2017-2018, the total 57,920 households produced 575 strata with an average of 86.5 households per strata (ranging 16-524 households).

The food intake information used in this study is based on the food purchases of each household for a period of seven consecutive days, registered by the respondents or by the survey interviewers in the booklet of collective expenditures (considering the common household measures or the unit of acquisition) and converted to kilograms or liters by the IBGE. The field data recollection for each survey was distributed along the four trimesters of the year, incorporating the seasonal variability of foods and expenditures.

The short reference period employed by the POF for recording household food expenditures in each household (seven days) does not allow for the identification of the usual food purchase patterns of each household. In this analysis, the cluster of households was used as study unit, corresponding to the set of households visited within each stratum in the sample of each household.

When necessary, the non-edible part of the gross quantity of each purchase by the household was excluded using correction factors estimated by IBGE. The edible part of each purchase in all surveys (2002-2003, 2008-2009, and 2017-2018) was converted into energy (kcal) and sodium (grams) using the Brazilian Food Composition Table, version 7.1 (TBCA, acronym in Portuguese), considering the average sodium content of each food category ^18^. For foodstuffs preserved with salt, such as beef jerky, dried and sun-dried meat, and salted fish, the equivalent quantity and sodium concentrate of the de-salted food was considered.

Food data were divided into food groups following the NOVA food classification: fresh and minimally processed foods, processed culinary ingredients, processed foods, and ultra-processed foods ^19^. The daily available energy and sodium per capita for each study unit (household stratum) were calculated. The available sodium was adjusted considering a total energy intake of 2,000 calories for the adult population.

Means of the availability of sodium in the POF 2017-2018 (and respective standard errors) are shown for all five regions of Brazil, and these means were disaggregated into urban and rural situations and for quintiles of per capita income distribution observed in the strata of this study. The contribution of each food group to the total sodium intake was evaluated according to quintiles of the distribution of household income. The trends for this share were evaluated using linear regression models, with the contribution of each food group in the household total sodium availability as an outcome and quintiles of distribution of income as explanatory variables. Values of p < 0.05 were considered significant.

The household sodium availability contribution of each food group is described for the country as a whole, comparing data from the POFs 2002-2003 and 2008-2009 to data from 2017-2018.

The software Stata, version 15 (https://www.stata.com), was used to analyze data, considering the sample weights of the study units.

## Results

In 2017-2018, the estimated sodium intake for each 2,000kcal was over 4g/day in all regions of Brazil and higher in strata of urban households compared to the rural areas. The lowest intakes were found in rural households in the Central-West region, and the highest, in urban households in the South. Additionally, the estimated sodium intake per 2,000kcal exceeded the WHO recommendation of 2g/day in all geographic regions of Brazil (Table 1).

**Table 1:** Household availability of energy and sodium, according to macroregion and rural or urban location. Brazil, 2017-2018.

Macroregion/Location	Sodium (g/person/2,000kcal)	
	Mean	SE
North		
Urban	4.3	0.2
Rural	4.1	0.2
Total	4.3	0.2
Northeast		
Urban	4.5	0.1
Rural	3.7	0.2
Total	4.3	0.1
Southeast		
Urban	4.8	0.1
Rural	4.1	0.2
Total	4.8	0.1
South		
Urban	5.8	0.1
Rural	4.4	0.2
Total	5.6	0.1
Central-West		
Urban	4.3	0.2
Rural	3.0	0.2
Total	4.2	0.2
Brazil		
Urban	4.8	0.1
Rural	3.9	0.1
Total	4.7	0.1

SE: standard error.

The estimations of sodium intake for each 2,000kcal gradually increased from 3.9g/day in 2002-2003 to 4.1g/day in 2008-2009, which later increased to 4.7g/day in 2017-2018. Additionally, over time, the estimated sodium intake for 2,000kcal increased in all income groups, although sodium intake remained inversely proportional with income increase, with a 27% difference between the highest and lowest quintile of income distribution per person for the Brazilian population (Table 2).

**Table 2:** Household availability of energy and sodium, according to quintiles of income distribution per person. Brazil, 2002-2003, 2008-2009, and 2017-2018.

Quintile of per capita income	Sodium g/person/2,000kcal						Trend coefficient	p-value *
	2002-2003		2008-2009		2017-2018			
	Mean	SE	Mean	SE	Mean	SE		
1st	3.4	0.1	3.9	0.1	4.0	0.1	0.0	0.252
2nd	3.6	0.1	3.9	0.1	4.5	0.1	0.1	0.015
3rd	3.8	0.1	4.0	0.1	4.9	0.1	0.1	0.128
4th	4.2	0.1	4.3	0.1	5.0	0.1	0.1	0.142
5th	4.5	0.1	4.5	0.2	5.1	0.2	0.0	0.237
Total	3.9	0.1	4.1	0.1	4.7	0.1	0.1	0.070

SE: standard error.

* p-value for linear trend among income quintiles per person.

**Figure 1:**
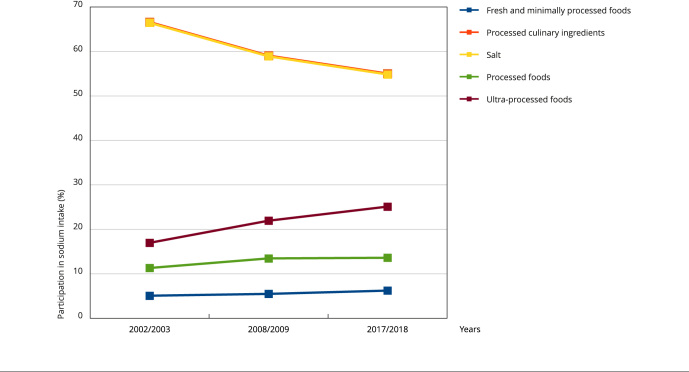
Participation of food groups in the total sodium intake of the Brazilian population, from 2002-2003 to 2017-2018.

Considering the dietary sources of sodium, in 2017-2018, the household purchases of processed culinary ingredients contributed to 55% of total sodium available for consumption (and specifically 54.9% from table salt), which shows a continuous decrease in their participation in comparison to 2008-2009 (59.1%) and 2002-2003 (66.6%). Meanwhile, processed foods contributed to 11.3% of sodium in 2002-2003, 13.5% in 2008-2009, and 13.6% in 2017-2018. Ultra-processed foods sodium contribution increased from 17% in 2002-2003 to 21.9% in 2008-2009 and then to 25.1% in 2017-2018. The participation of fresh and minimally processed foods in sodium availability increased less over time: 5.1% in 2002-2003 to 5.5% in 2008-2009 and finally 6.2% in 2017-2018 (5.9%) (Figure 1).

Table 3 shows that the contribution of processed culinary ingredients decreased over time in all income groups, yet its participation differs significantly according to household income, varying from 50% to 62.3% between the highest and lowest quintiles of income in 2017-2018. The contribution of ultra-processed foods increased significantly in all income groups from 2002-2003 to 2008-2009 but increased more intensely in poorer households from 2008-2009 to 2017-2018 (11.9% to 16.5%), while their participation in the highest income quintile had a small decline in the same period (31.4% to 29.7%).

**Table 3:** Distribution (%) of the household availability of sodium in quintiles of distribution of household income per person, for fresh and minimally processed foods and processed culinary ingredients. Brazil, 2002-2003, 2008-2009, and 2017-2018.

Food groups and subgroups	Brazil	Quintile of per capita income				
		1st	2nd	3rd	4th	5th
2002-2003						
Fresh and minimally processed foods	5.1	3.8	4.9	4.4	5.5	6.8 *
Processed culinary ingredients	66.6	76.3	72.0	70.1	61.1	53.1 *
Salt	66.5	76.2	71.9	70.0	60.9	52.8 *
Processed foods	11.3	11.0	9.8	9.4	11.9	14.6
Ultra-processed foods	17.0	8.9	13.3	16.1	21.5	25.5 *
2008-2009						
Fresh and minimally processed foods	5.5	4.1	4.9	6.3	5.6	6.5
Processed culinary ingredients	59.1	73.0	63.1	54.0	59.4	46.5 *
Salt	58.9	72.8	62.9	53.8	59.3	46.3 *
Processed foods	13.5	11.0	14.5	14.9	11.0	15.6
Ultra-processed foods	21.9	11.9	17.4	24.9	24.1	31.4 *
2017-2018						
Fresh and minimally processed foods	6.2	6.3	5.8	6.4	6.3	6.4
Processed culinary ingredients	55.0	62.3	56.0	54.2	52.5	50.0 *
Salt	54.8	62.1	55.8	54.0	52.4	49.6 *
Processed foods	13.6	14.9	15.4	11.5	12.3	14.0
Ultra-processed foods	25.1	16.5	22.8	27.9	28.8	29.7 *

* p-value < 0.05 for linear trend between quintiles of per capita income.

## Discussion

In this study, we used nationally representative data from 2002-2003 to 2017-2018, which allowed us to analyze historical trends in the population’s sodium intake and in its main dietary sources ^10^ ^,^ ^11^. The estimates of sodium available for consumption in Brazilian households remained above the maximum daily limit recommended by the WHO and increased from 2002-2003 to 2017-2018 (3.9g to 4.7g/day). Excessive availability of sodium is observed throughout Brazil, in both urban and rural areas and in all income strata, and the burden of excessive sodium intake on health outcomes, especially cardiovascular disease, has consequently increased over the last decades as well.

The only direct population representative estimate of the average sodium intake of adults in Brazil (3.7g/day) was obtained via spot urine samples in 2013, while data from personal food consumption, also from the POFs, estimated an average sodium intake of Brazilian adults of 2.5g/day in 2017-2018 ^12^. Our study estimated the average intake at 4.7g/day. The differences among these indirect estimates are mostly because dietary recalls use foods as consumed considering standardized recipes, while food acquisition surveys, as in this study, consider the number of culinary ingredients, especially salt and salt-based condiments, and other raw and minimally processed ingredients. Additionally, this study used the NOVA food classification for the analysis of food groups, while the study using national food records and recalls did not.

Information on sodium intake and dietary sodium sources are key to inform the creation and implementation of health policies. Despite the 24-hour urine samples being the gold standard for assessing sodium intake, the adoption of this methodology is not widespread due to its costs and logistics. Therefore, alternatively, indirect methods for sodium intake, such as estimates based on personal food consumption or household food acquisition, have also been used for estimating sodium intake by many countries, although they may, respectively, under- or overestimate the average sodium intake. Nevertheless, these indirect methods are very useful for identifying dietary sources of sodium ^20^ ^,^ ^21^ ^,^ ^22^.

Regarding dietary sources of sodium, processed culinary ingredients accounted for 55% of sodium in the diets of Brazilians in 2017-2018, yet its participation has decreased 17.4% since 2002-2003. Meanwhile, processed and ultra-processed foods have increased their participation as sodium sources in the diet, accounting for 20.3% and 47.6%, respectively. Together, they represented 38.7% of sodium in the diet in 2017-2018, compared to 28.3% in 2002-2003. Although the participation of ultra-processed foods is larger among higher income households over all periods analyzed (almost 1/3 of the sodium consumed), the participation of sodium from ultra-processed foods in diets of lower income households has almost doubled in the last 15 years, narrowing the gap among these income groups.

The changes in the dietary sources of sodium in Brazilian households is related to the gradual increase in the participation of processed and ultra-processed foods, particularly in lower income households, being less significantly attributed to the growth in the participation of sodium from fresh and minimally processed foods in all income groups ^14^.

This directly reflects the changes in the dietary patterns of the Brazilian population according to the extent and purpose of food processing. Homemade meals, based on the traditional diets and on local fresh ingredients, are gradually being replaced by ultra-processed foods, including ready to eat meals. This is also reflected in the decrease in the household purchases of table salt and salt-based condiments (culinary ingredients necessary for preparing homemade meals) and of staple foods in the Brazilian diet, such as rice and beans, together with an increase in the consumption of processed and ultra-processed foods, which hold higher sodium density than fresh and minimally processed foods ^14^.

Furthermore, sodium intake reduction is expected to occur by reducing added salt and salt-based condiments to foods together with reducing the sodium content and the consumption of processed and ultra-processed foods. Sodium intake can also be reduced by consuming fresh and minimally processed foods, which present lower sodium content and, thus, should be encouraged. The increased participation of ultra-processed foods to sodium intake highlights the importance of strengthening the current policies addressing sodium reduction, as recommended by the *Dietary Guidelines for the Brazilian Population* ^23^, and complementarily stimulating further food reformulation for reducing the sodium content of foods by industries.

Excessive sodium intake is a major risk factor for diet-related NCDs in most countries and must be considered a priority in health policies due to its health and economic burden. Moreover, sodium reduction policies are acknowledged as some of the most cost-effective policies for NCD prevention and control ^24^ ^,^ ^25^.

Dietary sources of sodium are diverse and may vary among countries and different population groups. For example, most sodium consumed in European countries, the United States, and Canada comes from industrialized foods. Meanwhile, in most Latin American countries, in Japan, and in China, the main sources of sodium are salt and salt-based condiments added to dishes ^26^.

Addressing the multiple sources of sodium in diets and changing historical trends in these sources, as in Brazil, requires multicomponent strategies for more effective policies. These strategies may include public awareness campaigns and education for both the general public and healthcare professionals; mandatory information on sodium content on food labels and front-of-package labelling schemes; mandatory or voluntary limits for sodium content in processed and ultra-processed foods; regulation of advertising and sales of foods with high sodium content, especially for children and adolescents; use of salt-substitutes; and taxation of foods with high sodium content ^25^.

Considering the policy implications of this study, especially in spite of the increase in the participation of processed and ultra-processed foods and the continuity of table salt as the major source of sodium in the diet, the national dietary guidelines provide direct recommendations. They address both the need to use small quantities of table salt and other culinary ingredients while cooking meals and the awareness of foods that are often high in sodium, such as processed and ultra-processed foods. Therefore, these guidelines should be the basis for communication and education strategies on sodium reduction ^23^.

Other initiatives to reduce sodium from processed and ultra-processed foods include the front of package labelling with warnings on high sodium content that was approved in 2020 ^27^ and the voluntary sodium targets that have been set since 2011 for key food categories ^28^ aiming to, respectively, increase consumer knowledge on the nutrient profile of foods and stimulate food industries to reduce the sodium content of products.

Regarding the national agreements with food industries for setting sodium targets, maximizing the policy impacts on sodium intake and on health outcomes will depend on broadening the targeted food categories, adopting regional and global benchmarks for sodium reduction ^29^ ^,^ ^30^, strengthening the existing official monitoring and enforcement mechanisms, and controlling the use of food additives (e.g., preservatives) and/or nutrients (sugars or fats) added in place of sodium. Moreover, independent monitoring and evaluation of strategies should be implemented to avoid possible conflicts of interest and, in the future, more stringent targets, based on mandatory approaches should increase the reach and the impact of sodium reduction ^31^ ^,^ ^32^ ^,^ ^33^. Nevertheless, food reformulation aimed solely to critical ingredients, such as sodium, is limited in reducing the overall risks of ultra-processed food consumption ^34^.

For added impact on salt reduction at the population level, new initiatives are also needed for addressing the commercialization of unhealthy foods in schools, advertising of unhealthy foods, and foods consumed out of home, including nutritional information on menus in food businesses, actions aimed at reducing the addition of sodium salts to foods prepared by food services, and perhaps sodium targets for foods sold in restaurants and fast foods, together with initiatives for reducing sodium in street foods and artisanal foods ^35^.

This study presents several strengths and limitations. Firstly, indirect estimates of sodium intake represent important tools for sodium reduction policies, especially regarding the dietary sources of sodium that should be prioritized, possible strategies to address them, and time trends in sodium intake estimates. The use of food purchases methods have been justified by the existing historical series of information on estimating the impact of added salt to diets and by recent evidence that concluded that, with few exceptions, food purchase expressed as relative energy contribution, as opposed to absolute weight, can provide a good picture of actual consumption in the Brazilian population ^20^ ^,^ ^36^.

Among the limitations of the study, the total sodium estimates from food acquisition may be overestimated and should ideally be completed by other data such as from dietary recalls and urinary sodium excretion ^20^. Most of this possible bias is caused by differences between sodium levels of foods in homemade meals and those eaten out of the households and the estimation of household table salt consumption and food waste. Nevertheless, food recalls are also subject to bias, especially in terms of measuring added salt to foods and assuming standardized recipes, so they generally underestimate sodium intake, while urinary sodium excretion studies tend to be more costly and have a more complex logistics. Additionally, food composition tables used for both food acquisition and food recalls are based on the average sodium content of each food category since data collection does not identify specific brands.

The efficacy of estimating sodium consumption every 2,000kcal/person/day relies on several assumptions. For example, that the meals eaten out of home are assumed to have similar sodium levels to homemade meals and that the fraction of food waste is independent of its sodium content. Moreover, the use of strata provides a better description of purchase patterns due to the short period of data collection; however, this may possibly introduce some bias to the analyses by artificially homogenizing the results.

The short reference period (one week) for collecting data on the purchase of elements in each household, according to the POF, may also represent a limitation of this study. To minimize this effect, homogenous groups of households, regarding location and socioeconomic characteristics (strata) were adopted as the unit of analysis, studied over 12 months. Another limitation of this study is the absence of individual-based dietary information, relying on an average distribution of foods among the family, and of information on food waste.

In conclusion, sodium intake in Brazil remains higher than daily recommendations in all macro regions and income classes. Over the last 15 years, the sodium intake by consumption of processed culinary ingredients, including table salt, showed a decreasing trend, whereas the proportion of sodium from processed and ultra-processed foods increased. Together, these dietary sources correspond to almost 94% of sodium available in the Brazilian households, so reducing population’s sodium intake in Brazil remains a major public health challenge that requires multiple strategies.
